# Hospital Readmissions in Patients with Severe Acute Respiratory Syndrome: A Retrospective Study in Northeastern Brazil

**DOI:** 10.3390/diseases14090308

**Published:** 2026-08-26

**Authors:** Raphael Omena Wanderley, Carmina Silva dos Santos, Karine Ferreira Agra, Francisco Pirauá Alves Gonçalves, Douglas Tenório Paes, Gabriel Borges de Brito, Gabriele Maria de Oliveira Lucena, Lucas de Carvalho Carriço, José Roberto da Silva Junior, Eduardo Jorge da Fonseca Lima

**Affiliations:** 1 Faculdade Pernambucana de Saúde (FPS), Recife 51150-001, PE, Brazil; raphaelow@gmail.com (R.O.W.); karine.agra@imip.org.br (K.F.A.); douglastpaes0@gmail.com (D.T.P.); gabrielborges81968@gmail.com (G.B.d.B.); gabioliveiralucena@outlook.com (G.M.d.O.L.); lucas.carrico778@gmail.com (L.d.C.C.); 2 Instituto de Medicina Integral Prof. Fernando Figueira (IMIP), Recife 50070-550, PE, Brazil; carmina.santos@fps.edu.br (C.S.d.S.); francisco.piraua@imip.org.br (F.P.A.G.); eduardojorge@imip.org.br (E.J.d.F.L.); 3 Department of Community Health Sciences, University of Manitoba, Winnipeg, MB R3E 0T6, Canada

**Keywords:** severe acute respiratory syndrome, hospital readmission, influenza vaccination, COVID-19, comorbidities, public health

## Abstract

Background/Objectives: Severe acute respiratory syndrome (SARS) remains an important cause of hospitalization and mortality, particularly among vulnerable populations. Understanding the factors associated with previous hospitalization may support more effective prevention and vaccination strategies. This study aimed to analyze the epidemiological profile of previous hospitalization among patients with SARS in the state of Pernambuco, Brazil. Methods: A quantitative, retrospective, cross-sectional study was conducted using data from the Influenza Epidemiological Surveillance System (SIVEP-Gripe), including 79,852 adult patients (≥18 years) notified with SARS in Pernambuco between 2021 and 2024. Sociodemographic, clinical, and vaccination-related variables were analyzed to identify factors associated with previous hospitalization. Results: The prevalence of previous hospital admission (readmission history) was 6.6%. Patients with previous hospitalization had a significantly higher median age compared to those without previous hospitalization (66 vs. 63 years; *p* < 0.001). The prevalence of previous hospitalization was higher among individuals with chronic comorbidities, particularly hematologic diseases (prevalence ratio [PR] = 1.95; 95% confidence interval [CI]: 1.51–2.51; *p* < 0.001) and asthma (PR = 1.67; 95% CI: 1.42–1.96; *p* < 0.001). Influenza vaccination was associated with a lower prevalence of previous SARS hospitalization (PR = 1.29; 95% CI: 1.03–1.61; *p* = 0.023). In contrast, COVID-19 vaccination status showed no significant association with previous hospitalization. A history of previous SARS hospitalization was associated with a higher prevalence of death in subsequent hospitalizations (PR = 1.09; 95% CI: 1.01–1.18; *p* = 0.022). Conclusions: Older adults and individuals with chronic comorbidities showed a higher prevalence of previous SARS hospitalization in Pernambuco. The findings show an association between influenza vaccination and a lower prevalence of previous SARS hospitalization and highlight the importance of post-discharge follow-up and epidemiological surveillance.

## 1. Introduction

Severe acute respiratory syndrome (SARS) is characterized by an influenza-like illness accompanied by signs of severity (e.g., oxygen saturation ≤ 94%, respiratory distress or tachypnea, worsening of the clinical picture, and hypotension) in the absence of another specific diagnosis [1]. Alternatively, SARS can be defined by the onset of acute respiratory failure during the seasonal period of the etiological agent [1]. The main risk factors include advanced age, lack of adequate immunization, chronic comorbidities (including heart disease, stroke, and diabetes mellitus), and isolated immunosuppression related to pre-existing clinical complications or prolonged viral shedding [2,3,4,5,6,7].

The clinical cases of SARS are associated with numerous viruses that preferentially infect the upper airways and may cause rhinopharyngitis or complications in other systems [8]. Furthermore, the prevalence of respiratory viruses varies according to seasonality and geographic location. SARS-CoV-2 is currently the most common pathogen; however, respiratory syncytial virus, influenza virus, adenovirus, and rhinovirus also play prominent roles in the pathological process [9,10]. Vaccination remains one of the most effective public health strategies to reduce the burden of severe respiratory infections, especially those caused by influenza and SARS-CoV-2, contributing to lower hospitalization and mortality rates [3,11,12,13,14,15,16]. However, despite advances in immunization coverage, severe cases and recurrent hospitalizations remain a challenge, particularly among vulnerable populations.

Respiratory viral diseases may be associated with hospital readmissions and substantial healthcare utilization [15,16,17]. In this context, investigating previous SARS hospitalizations may provide relevant epidemiological information on recurrent healthcare utilization. Moreover, hospital readmission may serve as an indicator of the effectiveness of prevention and control strategies, enabling adjustments and improvements in public healthcare [16]. Readmissions also represent a significant challenge for the healthcare system, leading to additional costs and bed and workforce overload, as well as negatively impacting the quality of life of patients by increasing the risk of complications and mortality [17].

Epidemiological surveillance data from before the COVID-19 pandemic indicated 40,294 cases of SARS in Brazil up to the 52nd epidemiological week of 2019. In the Northeast region of Brazil, 6612 cases of SARS were reported, with Pernambuco accounting for about 40% (2486) of all notifications in the region. In the first semester of 2020, at the beginning of the SARS-CoV-2 pandemic, SARS notifications in the state increased to an average of 2517 per month, representing a 13-fold rise in incidence [18].

Despite this, the literature remains scarce regarding studies quantifying readmission rates among patients with SARS. Determining the proportion of patients readmitted after hospital discharge may reveal weaknesses in follow-up care and in strategies aimed at reducing hospital readmission rates in Pernambuco. In this context, advanced age, chronic comorbidities, and socioeconomic conditions have been associated with hospital readmission after discharge [19]. Comorbidities such as diabetes mellitus and cardiovascular disease may compromise immune response and recovery capacity, whereas socioeconomic factors, such as limited access to post-discharge healthcare, may contribute to worse clinical outcomes. In patients with SARS due to COVID-19, oxygen saturation < 95%, respiratory failure, and bacterial co-infection were associated with a higher risk of hospital readmission [20].

Although previous studies have investigated hospital readmission following COVID-19 and other respiratory viral infections, evidence describing the epidemiological profile of individuals with a history of previous SARS hospitalization using large population-based surveillance databases remains limited, particularly in Brazil. A comprehensive understanding of SARS and its associated readmissions is essential for developing effective prevention and control strategies, particularly regarding vaccination policies and post-discharge surveillance. Therefore, this study aimed to analyze the history of previous SARS hospitalization among patients with SARS in the state of Pernambuco, investigating factors associated with previous SARS hospitalization, with particular attention to vaccination status and clinical comorbidities.

## 2. Methods

This retrospective registry-based cross-sectional study used data from the Influenza Epidemiological Surveillance System (SIVEP-Gripe) to analyze the history of previous SARS hospitalization among patients with severe acute respiratory syndrome (SARS) in the state of Pernambuco, Brazil, from 2021 to 2024. The study included adult patients (≥18 years) admitted to the Brazilian Unified Health System (SUS) network with a confirmed notification of SARS, whose records were subsequently assessed for previous hospitalization history.

Access to the SIVEP-Gripe database was granted by the Pernambuco State Health Department. The study period (2021–2024) was selected because it encompasses the COVID-19 pandemic and post-pandemic periods, characterized by a higher burden of SARS cases and improved notification completeness. The study was approved by the Research Ethics Committee of Faculdade Pernambucana de Saúde (CAAE: 88854325.8.0000.5569; opinion No. 7,591,401). Informed consent was waived due to the retrospective design and use of anonymized secondary data.

After database consolidation into a single electronic spreadsheet, records from individuals aged under 18 years and variables unrelated to the study objectives (e.g., personal, administrative, and sanitary management data) were excluded. Incomplete records for the variables of interest were also excluded from the respective analyses.

The following variables were selected based on their clinical relevance and evidence from previous literature: sex, age, cardiovascular disease, hematologic disease, Down syndrome, asthma, diabetes mellitus, chronic lung disease, obesity, immunosuppression, influenza vaccination in the most recent campaign, COVID-19 vaccination status, clinical outcome, and history of previous hospitalization. Obesity was available only as a binary variable, without data for BMI calculation. Immunosuppression corresponded to the predefined SIVEP-Gripe field indicating the presence of immunodeficiency or immunosuppression, without information on its underlying cause, while hypertension was included within the broader category of chronic cardiovascular disease.

Previous SARS hospitalization was identified through deterministic longitudinal linkage of SIVEP-Gripe records using SHA-256 encrypted patient names and dates of birth. The linkage was conducted by sorting the eight-digit hash values alphanumerically and comparing each value to its adjacent neighbor. Because the SHA-256 algorithm generated these hashes solely based on patient names, dates of birth were also cross-referenced. This step significantly reduced the risk of misclassifying distinct patients with the same name as a single individual. The matching operation was performed unidirectionally so that only subsequent instances, but not the index case, would be accounted for. Notifications occurring less than 3 days apart were considered part of the same episode, to minimize duplicate records or inter-hospital transfers. Only SARS hospitalizations recorded in SIVEP-Gripe were considered. As no predefined follow-up interval was adopted, the outcome represents a history of previous SARS hospitalization rather than clinical readmission.

Data were analyzed using IBM SPSS Statistics version 25.0 (IBM Corp., Armonk, NY, USA) and Microsoft Excel 365. Descriptive analyses were presented as absolute and relative frequencies for categorical variables and as measures of central tendency and dispersion for continuous variables. Normality of age distribution was assessed using the Shapiro–Wilk test and Q–Q plots. Comparisons between groups with and without a history of previous SARS hospitalization were performed using the Mann–Whitney U test for age and the Chi-square test for categorical variables.

Crude prevalence ratios (PRs) and their 95% confidence intervals (95% CIs) were calculated using Epi Info. For most variables, PRs compared the presence (“Yes”) with the absence (“No”) of the characteristic, whereas the vaccinated group was the reference category for influenza and COVID-19 vaccination. Missing, ignored, and unknown values were excluded from each analysis without imputation, resulting in variable denominators. Statistical significance was set at *p* < 0.05.

## 3. Results

A total of 79,852 adult patients with severe acute respiratory syndrome (SARS) in Pernambuco were analyzed between 2021 and 2024, of whom 50.2% were male. Age ranged from 18 to 117 years, with a median of 63 years (interquartile range: 49–76). Among patients with available data, cardiovascular disease (28.8%) and diabetes mellitus (22.1%) were the most frequent comorbidities, followed by obesity (5.2%), chronic lung disease (2.3%), asthma (2.2%), immunosuppression (1.4%), hematologic disease (0.6%), and Down syndrome (0.1%) (Table 1).

Among the 11,657 patients with available information on influenza vaccination, 16.3% had received the vaccine in the most recent campaign. Regarding COVID-19 vaccination, 69.5% of the 31,193 patients with valid records had received at least one dose. Clinical outcome data were available for 46,754 patients, among whom 59.8% recovered and 40.2% died, including 39.0% from respiratory causes and 1.2% from other causes. Previous hospitalization for SARS was identified in 6.6% of the total sample (Table 2).

No significant association was observed between sex and previous hospitalization (*p* = 0.302). However, age was significantly associated with readmission, with patients who had previous hospitalizations presenting a higher median age compared with those without previous hospitalization (66 vs. 63 years; *p* < 0.001) (Table 3).

Regarding clinical factors, cardiovascular disease was associated with a higher prevalence of previous hospitalization (7.1% vs. 5.8%; PR = 1.24; 95% CI: 1.12–1.38; *p* < 0.001). Similar associations were observed for hematologic disease (11.3% vs. 5.8%; PR = 1.95; 95% CI: 1.51–2.51; *p* < 0.001), asthma (9.6% vs. 5.8%; PR = 1.67; 95% CI: 1.42–1.96; *p* < 0.001), diabetes mellitus (7.3% vs. 5.6%; PR = 1.30; 95% CI: 1.17–1.44; *p* < 0.001), and chronic lung disease (9.0% vs. 5.8%; PR = 1.55; 95% CI: 1.32–1.82; *p* < 0.001). No significant associations were found for Down syndrome, obesity, or immunosuppression (Table 3).

Influenza vaccination was associated with a lower prevalence of previous hospitalization, with vaccinated patients presenting a readmission rate of 4.5% compared with 5.8% among unvaccinated individuals (PR = 1.29; 95% CI: 1.03–1.61; *p* = 0.023). In contrast, COVID-19 vaccination status was not significantly associated with previous hospitalization (*p* = 0.488) (Table 4).

Regarding clinical outcomes, patients who died had a slightly higher prevalence of previous hospitalization than those who recovered (6.0% vs. 5.5%; PR = 1.09; 95% CI: 1.01–1.18; *p* = 0.022) (Table 4).

## 4. Discussion

The present study analyzed a large population-based database from Pernambuco and identified a 6.6% prevalence of previous hospitalization among patients with severe acute respiratory syndrome (SARS), highlighting a clear vulnerability profile characterized by older age and chronic comorbidities. These findings are consistent with national and international literature and reinforce the relevance of immunization strategies and epidemiological surveillance in the management of severe respiratory infections [6,15,16,20]. These findings reflect a history of previous SARS hospitalization rather than clinical readmission, as no predefined follow-up interval was established.

The balanced sex distribution observed among SARS cases in Pernambuco may reflect the broad dissemination of respiratory viruses during the pandemic and post-pandemic periods, as well as relatively homogeneous access to health services in the region. Although no differences in readmission were observed between males and females, previous studies have shown that male patients may experience greater severity and worse clinical outcomes, possibly due to biological and behavioral factors [21]. Therefore, the similar prevalence of previous SARS hospitalization between sexes should not be interpreted as evidence of similar clinical outcomes or prognosis.

Older age was significantly associated with previous hospitalization, reinforcing its role as a major risk factor for severe respiratory outcomes. This finding is in agreement with previous studies showing that frailty, multimorbidity, and polypharmacy contribute to worse prognosis and higher hospitalization rates among older adults [22]. The higher median age among patients with previous hospitalization in our sample supports the hypothesis that immunosenescence and reduced physiological reserve may increase susceptibility to recurrent severe episodes. Age may also confound the observed associations due to its relationship with comorbidity burden and should therefore be considered when interpreting these findings.

Although COVID-19 vaccination was not associated with a history of previous SARS hospitalization in our study, extensive evidence has demonstrated its effectiveness in preventing severe COVID-19 and hospitalization before the onset of critical illness [23]. The absence of a statistically significant association may reflect the high vaccination coverage in Pernambuco and characteristics of the surveillance data, and should therefore be interpreted with caution. This finding should not be interpreted as evidence of reduced vaccine effectiveness. A large multicenter cohort in the United States similarly found that vaccination was not an independent predictor of hospital readmission but was associated with lower mortality among readmitted patients [24].

Comorbidities were strongly associated with previous hospitalization, particularly hematologic disease, asthma, diabetes mellitus, cardiovascular disease, and chronic lung disease. These findings are consistent with previous evidence showing that multimorbidity increases the risk of hospital readmission in patients with respiratory diseases [6,25]. Chronic diseases may impair immune response, reduce recovery capacity, and increase vulnerability to recurrent severe infections, which may explain the observed associations.

In contrast, Down syndrome, obesity, and immunosuppression were not significantly associated with previous hospitalization in our sample. This differs from previous reports identifying obesity and immunosuppression as prognostic factors for severe COVID-19 outcomes [26]. These discrepancies may be related to differences in study populations, disease severity, and outcome definitions. Regarding Down syndrome, the low frequency observed in our sample may have limited the statistical power to detect associations, particularly considering its greater clinical relevance in younger populations [27].

Another important finding was the association between previous hospitalization and mortality. Patients who died had a slightly higher prevalence of previous hospitalization than those who recovered, suggesting that recurrent severe respiratory episodes may indicate a higher degree of clinical frailty and vulnerability. This interpretation is supported by studies showing overlap between risk factors for readmission and mortality [28,29]. In this context, previous hospitalization may represent an important marker for intensified post-discharge surveillance and preventive care.

From a clinical and public health perspective, the higher prevalence of previous SARS hospitalization among older adults and individuals with chronic comorbidities highlights the importance of recognizing these groups in post-discharge care and epidemiological surveillance. These findings may support strategies focused on continuity of care, monitoring after severe respiratory episodes, and maintenance of vaccination programs. At the population level, identifying patterns associated with previous SARS hospitalization may also contribute to surveillance planning and resource allocation, particularly during periods of increased circulation of respiratory viruses. Future longitudinal studies with clearly defined follow-up periods, more detailed information on vaccination and comorbidity burden, and multivariable analyses are needed to identify independent predictors of subsequent SARS hospitalization and to evaluate the effectiveness of post-discharge preventive strategies.

This study has important strengths, including the large population-based sample and the use of a national surveillance system covering SARS cases over a four-year period. The longitudinal linkage of SIVEP-Gripe records also enabled the identification of previous SARS hospitalizations within this large surveillance dataset. However, several limitations should be considered. The high and heterogeneous proportion of missing data, particularly for comorbidities and vaccination status, required a complete-case approach with varying denominators across analyses, which may have reduced precision and introduced information and selection bias. Consequently, the analyzed subgroups may not fully represent the overall study population. The substantial amount of missing data also precluded reliable multivariable adjustment. Therefore, the reported associations are unadjusted and may be affected by residual confounding, particularly by age and coexisting comorbidities. Limited information on disease severity, intervals between hospitalizations, post-discharge follow-up, and COVID-19 vaccination should also be considered when interpreting the findings.

## 5. Conclusions

The prevalence of previous hospitalization for severe acute respiratory syndrome (SARS) in Pernambuco was considerable and disproportionately affected older adults and individuals with chronic comorbidities. The findings reinforce influenza vaccination as an essential protective strategy and indicate that a history of previous hospitalization was associated with higher mortality.

These results highlight the importance of implementing post-discharge follow-up protocols and strengthening annual influenza vaccination campaigns, especially among more vulnerable groups. In addition, improving epidemiological surveillance and strengthening primary healthcare may contribute to the early identification of vulnerable patients, better disease monitoring, and a reduction in recurrent hospitalizations and their burden on the healthcare system.

## Figures and Tables

**Table 1:** Sociodemographic and clinical characteristics of adult patients with severe acute respiratory syndrome (SARS) in Pernambuco, Brazil, 2021–2024.

Variables	*n*	Overall (%)	Valid (%)
Sex			
Male	40,049	50.1	50.2
Female	39,755	49.8	49.8
Missing	48	0.1	—
Cardiovascular disease			
Yes	22,980	28.8	76.4
No	7115	8.9	23.6
Missing	49,757	62.3	—
Hematologic disease			
Yes	512	0.6	4
No	12,248	15.4	96
Missing	67,092	84	—
Down syndrome			
Yes	116	0.1	0.9
No	12,330	15.4	99.1
Missing	67,406	84.5	—
Asthma			
Yes	1760	2.2	12.9
No	11,844	14.8	87.1
Missing	66,248	83	—
Diabetes mellitus			
Yes	17,619	22.1	70.3
No	7457	9.3	29.7
Missing	54,776	68.6	—
Chronic lung disease			
Yes	1827	2.3	13.5
No	11,730	14.7	86.5
Missing	66,295	83	—
Obesity			
Yes	4190	5.2	27.5
No	11,024	13.8	72.5
Missing	64,638	80.9	—
Immunosuppression			
Yes	1140	1.4	8.8
No	11,874	14.9	91.2
Missing	66,838	83.7	—

Abbreviations: P25, 25th percentile; P75, 75th percentile. Data source: Brazilian Influenza Epidemiological Surveillance Information System (SIVEP-Gripe).

**Table 2:** Vaccination status, clinical outcomes, and hospitalization history of adult patients with severe acute respiratory syndrome (SARS) in Pernambuco, Brazil, 2021–2024.

Variables	*n*	Overall (%)	Valid (%)
Influenza vaccine in the most recent campaign			
Yes	1898	2.4	16.3
No	9759	12.2	83.7
Missing	68,195	85.4	—
COVID-19 vaccine			
Yes	21,694	27.2	69.5
No	9499	11.9	30.5
Missing	48,659	60.9	—
Clinical outcome			
Recovery	27,999	35.1	59.8
Death	18,216	22.8	39
Death from other causes	539	0.7	1.2
Missing	33,098	41.4	—
Previous hospitalization			
Yes	5296	6.6	6.6
No	74,556	93.4	93.4

Data source: Brazilian Influenza Epidemiological Surveillance Information System (SIVEP-Gripe).

**Table 3:** Factors associated with previous hospitalization among adult patients with severe acute respiratory syndrome (SARS) in Pernambuco, Brazil, 2021–2024.

Variables	Yes *n* (%)	No *n* (%)	PR	95% CI	*p*-Value
Sex					
Male	2621 (6.5)	37,428 (93.5)	1	—	0.302 *
Female	2674 (6.7)	37,081 (93.3)	1.03	0.98–1.08	
Cardiovascular disease					
Yes	1643 (7.1)	21,337 (92.9)	1.24	1.12–1.38	< 0.001 *
No	410 (5.8)	6705 (94.2)	1	—	
Hematologic disease					
Yes	58 (11.3)	454 (88.7)	1.95	1.51–2.51	< 0.001 *
No	713 (5.8)	11,535 (94.2)	1	—	
Down syndrome					
Yes	10 (8.6)	106 (91.4)	1.47	0.81–2.67	0.209 *
No	723 (5.9)	11,607 (94.1)	1	—	
Asthma					
Yes	169 (9.6)	1591 (90.4)	1.67	1.42–1.96	< 0.001 *
No	682 (5.8)	11,162 (94.2)	1	—	
Diabetes mellitus					
Yes	1285 (7.3)	16,334 (92.7)	1.3	1.17–1.44	< 0.001 *
No	419 (5.6)	7038 (94.4)	1	—	
Chronic lung disease					
Yes	165 (9.0)	1662 (91.0)	1.55	1.32–1.82	< 0.001 *
No	684 (5.8)	11,046 (94.2)	1	—	
Obesity					
Yes	261 (6.2)	3929 (93.8)	1.07	0.93–1.23	0.323 *
No	640 (5.8)	10,384 (94.2)	1	—	
Immunosuppression					
Yes	60 (5.3)	1080 (94.7)	0.89	0.69–1.15	0.385 *
No	700 (5.9)	11,174 (94.1)	1	—	
Variables	Previous hospitalization	No previous hospitalization			*p*-value
Age (years), median (P25–P75)	66.0 (52.0–77.0)	63.0 (49.0–76.0)			< 0.001 **

Abbreviations: PR, prevalence ratio; CI, confidence interval; P25, 25th percentile; P75, 75th percentile. Reference categories are indicated by PR = 1.00. * Chi-square test. ** Mann–Whitney U test. Data source: Brazilian Influenza Epidemiological Surveillance Information System (SIVEP-Gripe).

**Table 4:** Association among vaccination status, clinical outcomes, and hospitalization history with previous hospitalization among adult patients with severe acute respiratory syndrome (SARS) in Pernambuco, Brazil, 2021–2024.

Variables	Yes *n* (%)	No *n* (%)	PR	95% CI	*p*-Value
Influenza vaccine in the most recent campaign					
Yes	86 (4.5)	1812 (95.5)	1	—	0.023 *
No	570 (5.8)	9189 (94.2)	1.29	1.03–1.61	
COVID-19 vaccine					
Yes	1388 (6.4)	20,306 (93.6)	1	—	0.488 *
No	588 (6.2)	8911 (93.8)	0.97	0.88–1.06	
Clinical outcome					
Recovery	1528 (5.5)	26,471 (94.5)	1	—	0.022 *
Death	1117 (6.0)	17,638 (94.0)	1.09	1.01–1.18	

Abbreviations: PR, prevalence ratio; CI, confidence interval. * Chi-square test. Data source: Brazilian Influenza Epidemiological Surveillance Information System (SIVEP-Gripe).

## Data Availability

The data presented in this study were derived from the Influenza Epidemiological Surveillance System (SIVEP-Gripe). Access to the data was granted by the Pernambuco State Health Department. The data are available from the corresponding author upon reasonable request and with permission of the Pernambuco State Health Department.
